# COVID-19 and Mitochondrial Non-Coding RNAs: New Insights From Published Data

**DOI:** 10.3389/fphys.2021.805005

**Published:** 2022-02-04

**Authors:** Andrea Pozzi

**Affiliations:** Graduate School of Life Sciences, Tohoku University, Sendai, Japan

**Keywords:** non-coding RNAs, mitochondria, mitochondrial metabolism, COVID-19, long COVID, SARS-CoV-2

## Abstract

Scientists all around the world are working to investigate new ways to prevent and treat COVID-19, and recent research has been focusing on the effects of a syndrome commonly called “long COVID.” People affected by this syndrome usually suffer from symptoms like the ones observed in several types of fatigue syndrome. As these syndromes are often linked to mitochondrial dysfunction, researchers hypothesized that a dysfunction in the mitochondrial metabolism might be part of the causes of long COVID. However, while there are a few studies investigating the effect of SARS-CoV-2 infection on mitochondrial metabolism, the effect on the transcription of mitochondrial non-coding RNAs has not been investigated yet. Thus, using publicly available data, I explored the effect of SARS-CoV-2 on the expression of several mitochondrial non-coding RNAs in patients recovering from COVID-19. No change in the expression of long non-coding RNAs was detected at any stage of the infection, but up to 43 small mitochondrial RNAs have their expression altered during the recovery from COVID-19. This result suggests that the SARS-CoV-2 infection somehow affected the metabolism of small mitochondrial RNAs specifically without altering the overall mitochondrial transcription. Despite these being only preliminary results on a small cohort, the analyses clearly showed that individuals infected by SARS-CoV-2 retain an altered expression of these small RNAs. This persistent alteration in the expression of small mitochondrial RNAs might be involved in the long COVID syndrome and further studies are needed to confirm the possibility.

## Introduction

Although the majority of individuals fully recover from SARS-CoV-2 infections, sometimes a subgroup of patients develops a condition generally known as “long COVID syndrome” where the patient still has lingering symptoms from the infection. Although the full clinical features of the “long COVID syndrome” are not known yet (Komaroff and Bateman, 2020), many of its symptoms generally overlap with the ones observed in myalgic encephalomyelitis/chronic fatigue syndrome (ME/CFS; Paul et al., 2021). ME/CFS is a long-term debilitating illness that causes multiple symptoms, including extreme tiredness, sleep problems, and muscle pain. Although ME/CFS cause is unknown, a wide range of risk factors such as allergies or anxiety disorders (Hempel et al., 2008; Lievesley et al., 2014) has been identified. Among those, the oldest known potential cause of these symptoms is a viral infection. Indeed, when symptoms similar to the ME/CFS manifest after a viral infection, the condition is usually called “postviral fatigue syndrome,” or other names such as Iceland disease or Royal Free disease (Behan and Behan, 1988; Eriksen, 2018). The postviral fatigue syndrome matches what is observed seen in people suffering from long COVID syndrome, suggesting that long COVID syndrome might simply be the most recent case of postviral fatigue syndrome.

While the postviral fatigue syndrome has been known for decades, the mechanism used by viruses to cause this syndrome is still unknown. However, a few studies identified the disruption of the mitochondria metabolic pathway as one of the possible causes of the syndrome (Behan et al., 1991; Tomas et al., 2017). These studies led researchers to hypothesize that the SARS-CoV-2 infection might lead to a redox imbalance, similar to what was observed in postviral fatigue syndrome, thus causing the symptoms observed in the “long COVID” syndrome (Komaroff and Bateman, 2020; Paul et al., 2021). A few recent studies support this hypothesis. For example, researchers found that SARS-CoV-2 infection can lower the expression of nuclear-encoded genes related to the mitochondrial Complex I (Miller et al., 2021), thus disrupting the mitochondrial function in a similar way to what was observed in patients suffering from postviral fatigue syndrome (Behan et al., 1991). However, the mechanism used by SARS-CoV-2 to disrupt mitochondrial metabolism is unknown. One possible mechanism used could be to alter the expression of genes encoded in the mitochondrial genome. This possibility has been already partially investigated, and a recent study showed that the expression of canonical mitochondrial genes (i.e., protein-coding genes involved in ATP production) is not impaired during COVID-19 (Miller et al., 2021). Nonetheless, this study focused on the canonical mitochondrial genes while not reporting on the expression of non-canonical genes encoded within the mitochondrial genome.

Most genes encoded within the human mitochondrial genome have been known for decades, however, a few new genes have been discovered only in the last decade. The well-known genes, here called “canonical genes,” are involved in ATP production (13 protein-coding genes, 22 tRNAs, and 2 rRNAs), while the newly discovered genes, here called “non-canonical genes,” are a mix of small peptides and non-coding RNAs which biogenesis and function are still not fully understood. Despite their recent discovery, some information is already known for a few non-canonical genes. For example, a recent study showed that double-stranded mitochondrial RNAs trigger antiviral signaling in humans and that this signaling is partially regulated by proteins such as PNPase and SUV3 (Dhir et al., 2018). Similarly, several long non-coding RNAs (mt-lncRNAs) encoded within the mitochondrial genome, encoded in the protein-coding genes CYTB, ND5, and ND6, are likely involved in mitochondrial metabolism and have been shown to be part of known RNA-biogenesis pathways (Rackham et al., 2011; Zhao et al., 2018; Statello et al., 2021). Another group of non-canonical mitochondrial genes that might be relevant for human health is small RNAs (mt-sRNAs), which presence and tissue-specific expression have been demonstrated across multiple organisms, ranging from clam to humans (Mercer et al., 2011; Ro et al., 2013; Pozzi et al., 2017; Pozzi and Dowling, 2019; Riggs et al., 2019). These small RNAs (25–35 nt) function is unknown, however, researchers hypothesized that they might influence gene expression of both nuclear and mitochondrial genes (Pozzi and Dowling, 2021). Despite not being well characterized, it is known that at least one group of the non-canonical mitochondrial genes is involved in the immune response again viruses (double-stranded RNAs) thus making it plausible for other types of mitochondrial non-coding RNAs to be involved as well (Dhir et al., 2018). In order to obtain some preliminary results on whether other non-canonical mitochondrial genes are involved in immune response and if these genes could be linked to the long COVID syndrome, I analyzed the data from a published study using samples from patients at different stages of COVID-19 recovery (Zheng et al., 2020).

A previously published RNA dataset was analyzed to investigate the role of non-canonical mitochondrial genes in long COVID, in this dataset both long and small RNAs have been sampled from the same 18 individuals of Chinese ethnicity during their recovery from a SARS-CoV-2 infection (Zheng et al., 2020). In the published study, the researchers split the patients into three categories during their COVID-19 recovery, treatment, convalescence, and rehabilitation. Their research focused on understanding the changes in expression of known micro RNAs and nuclear mRNAs, while not reporting on mitochondrial sequences. Thus, this study will be complementary, as it will focus only on mitochondrial sequences. As studies publishing samples with double-stranded RNAs from patients infected with COVID-19 are not available, this study focuses only on long and small RNAs (mt-lncRNAs and mt-sRNAs). The hypothesis is that because of SARS-CoV-2 on mitochondrial metabolism (Miller et al., 2021), mitochondria non-canonical genes expression might be disrupted at some stage of the infection. To test this hypothesis, the following rationale will be used. Usually, changes in the expression of a gene of interest during a specific event, such as an infection, can be explained either by the gene being involved in that event, such as immune response, or that the gene expression is disrupted because of the event. Furthermore, to verify if the changes in mitochondrial non-canonical genes are reliable, another small RNA dataset from a European population has been used, where a comparison between individuals suffering from COVID-19 and healthy individuals. By using the datasets available, it can be tested if mt-lncRNAs and mt-sRNAs expression changes at any, or all, stages of COVID-19 recovery, which would suggest that these genes are either involved in the response to SARS-CoV-2 infection or disrupted by its presence (or both), thus suggesting a possible link to long COVID-19 syndrome. Furthermore, by performing these tests, it is possible to obtain several insights into the effect of SARS-CoV-2 infection on mitochondrial expression.

## Results

**FIGURE 1:**
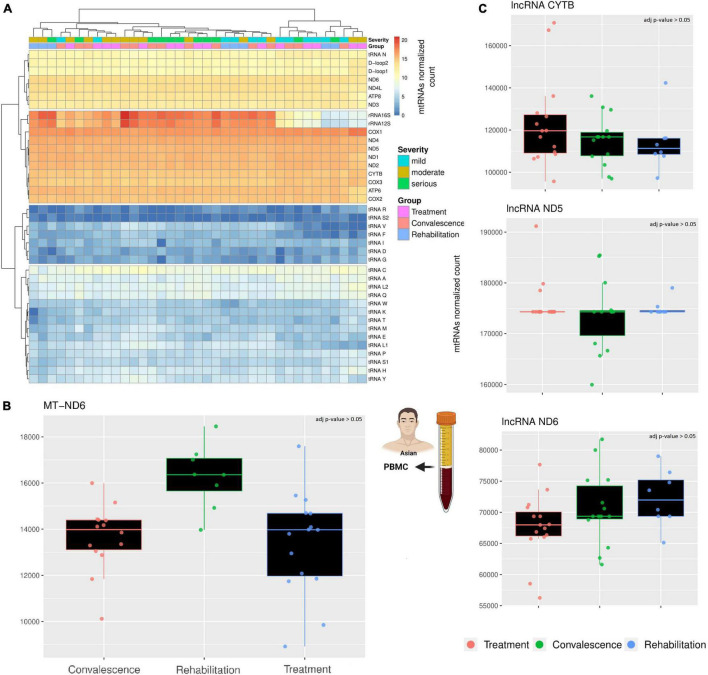
A comparison across three clinical stages of long RNAs expression. **(A)** The heatmap compare the expression of canonical genes encoded on the mitochondrial genome. The rows represent mitochondrial genes, while the columns are individuals. Each individual has annotated the severity of the COVID-19 infection and the stage when their blood sample was taken. According to the different level of expression, ranging from blue to red, four clusters can be identified. Both columns and rows have been clustered. **(B)** The barplot compare the expression of MT-ND6 mRNA across three clinical stages. Each datapoint in a single clinical stage (e.g., Treatment) represent a different patient, furthermore, the patients are the same across the three clinical stages. **(C)** The barplots compare the expression of three lncRNAs across three clinical stages using the same criteria of the previous barplot. The icons in the figure highlights from which population and type of sample the data are generated. The icons have been made using Biorender.

The first analysis focused on the general expression of the mitochondrial canonical genes across the three groups of patients (Figure 1A). These genes clustered in four expression clusters, with most mt-tRNAs staying in the two clusters with very low expression levels. This is expected, as the RNA library used for this analysis focuses mostly on longer RNAs (200 nt >) while mt-tRNAs are usually relatively short (∼70 nt). Similarly, the cluster with the highest expression includes the genes we expected to see: the two rRNAs and most protein-coding genes. However, the cluster with a moderate level of transcription includes a mix of genes, including regions without genes (D-Loop), a mt-tRNA (Asparagine), and a few protein-coding genes. Importantly, apart from MT-ND6, none of the canonical genes is differentially expressed across the three groups of patients (Figure 1B). Contrary to the others, the transcription of MT-ND6 seems stable during the first two clinical stages (treatment and convalescence) but is significantly upregulated during the rehabilitation stage (*p*-value < 0.05, Wald test adjusted with Benjamin-Hochberg correction). While these results mostly align with previously observed as we did not observe a change in mRNAs for most genes (Miller et al., 2021), the upregulation of ND6 after the infection has not been observed before and it supports the hypothesis that mitochondrial metabolism might be present in patients with long COVID (Komaroff and Bateman, 2020; Paul et al., 2021).

Like the canonical genes, the transcriptional profile of the three chosen lncRNAs is stable across clinical stages (Figure 1C). Indeed, a differential expression analysis confirmed the absence of any significant difference among the three clinical stages (*p*-value > 0.05, Wald test adjusted with Benjamin-Hochberg correction). Interestingly, the gene MT-ND6 harbor both a canonical gene (protein ND6) and a non-canonical gene (lncRNA ND6), but only the canonical gene is differentially expressed across the clinical stages. This difference suggests that the SARS-CoV-2 infection affects the different classes of mitochondrial RNAs independently.

**FIGURE 2:**
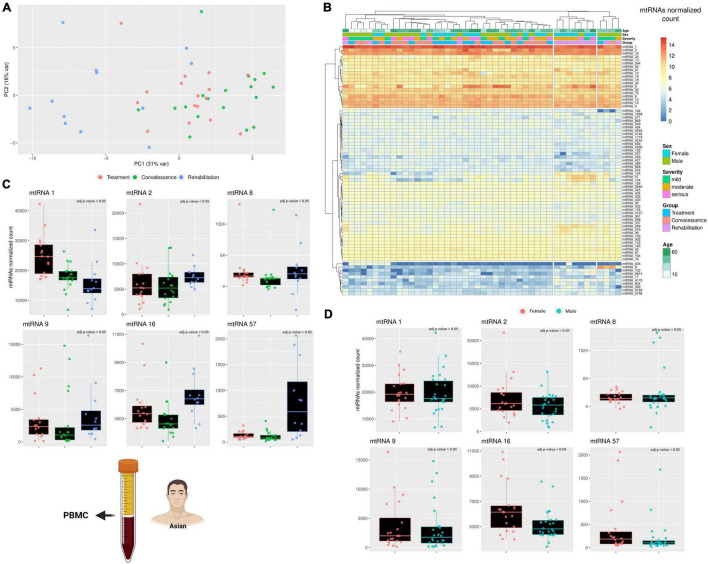
A comparison across three clinical stages of small RNAs expression. **(A)** The PCA shows how the differences in the small mitochondrial RNAs expression cluster the patients during the rehabilitation stages away from the other two clinical stages. This effect is evident already on the PC1, suggesting that only a small number of small mitochondrial RNAs is driving this effect. The variability of each principal component is in the axis label. **(B)** The heatmap compare the expression of small RNAs encoded on the mitochondrial genome. The rows represent clusters of small RNAs (mtRNAs), while the columns are individuals. Each individual has annotated the severity of the COVID-19 infection and the stage when their blood sample was taken, its age, and its sex (male/female). The different level of expression, ranging from blue to red. **(C,D)** The barplot compare the expression of several mtRNAs that are differentially transcribed across the three clinical stages **(C)** and across sexes **(D)**. Each datapoint in a single clinical stage (e.g., Rehabilitation) represent a different patient, furthermore, the patients are the same across the three clinical stages. Full stats can be found in Supplementary Table 1. Due to the presence of an outlier with very high expression, only mtRNA8 has its Y-axis in Log scale. The icons in the figure on the left-side highlights from which population and type of sample the data are generated. The icons have been made using Biorender.

After analyzing the longer RNAs, the focus of the analysis shifted on the other non-canonical genes, the small mitochondrial RNAs (mt-sRNAs). However, contrary to the mt-lncRNAs, it was not possible to choose mt-sRNAs previously characterized to analyze, considering that human mt-sRNA with known function or biogenesis are not known. Thus, the relevant mt-sRNAs were identified using a computational pipeline that create an assembly of these mt-sRNAs and then cluster partially overlapping RNAs into clusters. For simplicity, these clusters will be referred as “mtRNA” followed by an arbitrary number. Despite the clusters including slightly different sequences, each mtRNA has a “most expressed sequence” that can be found in the material and methods section. To visualize if these mt-sRNA clusters are different across the three clinical stages, a PCA analysis was performed (Figure 2A). The PCA analysis clustered together with the individuals during the treatment and convalescence but separated most of the individuals during the rehabilitation stage. This is particularly evident on the first component (PC1 31%) where eight of 12 individuals cluster together. Surprisingly, while rehabilitation is more distant temporarily from the treatment stage than to the convalescence stage, the PCA analysis shows that the expression profiles of patients in the rehabilitation and treatment are more similar than rehabilitation and convalescence. The differences across mt-sRNA clusters and stages become evident when performing a differential expression analysis (Figure 2B). The analysis includes a total of 75 mtRNAs (mt-sRNA clusters) which were selected with arbitrary parameters (having at least 50 reads in four different samples) meant to limit the number of clusters with very low expression or high expression only in one patient. Comparing the expression of the cluster across different degrees of COVID-19 infection severity (mild, moderate, and serious) did not yield any significant results. Statistics of the comparisons between clinical stages and sexes are available in Supplementary Table 1. Similarly, the comparison across different ages did not yield any significant results, potentially because most of the individuals were over 50 with only a few outliers (2 male children). However, when comparing these 75 mtRNAs across the three clinical stages, and across sexes, it became obvious that some mt-sRNA clusters have differential expression. In the comparison between males and females 19 mtRNAs are differentially expressed (*p*-value < 0.05, Wald test adjusted with Benjamin-Hochberg correction), although it is possible that the difference in age distribution across the two sexes might have affected the result. Likewise, across the three clinical stages, there are nine are differentially expressed between the stages of treatment/convalescence, while 33 and 43 mtRNAs are differentially expressed between the stage of treatment/rehabilitation and convalescence/rehabilitation, respectively (*p*-value < 0.05, Wald test adjusted with Benjamin-Hochberg correction). The mtRNAs changes in expression are different across the mtRNAs, with some being upregulated and some downregulated, suggesting that the increase in mtDNA copy number is not the cause of these changes. For example, the mtRNAs 144, 57, and 104 are upregulated during the rehabilitation stage, while others like mtRNA 1 are lower during the same stage. This mixed pattern suggests that these changes are caused by a specific regulatory pathway that affects these mtRNAs rather than a simple increase or decrease of mtDNA copy number, and this process might be linked to sex-specific differences. Indeed, some mtRNAs (including the three just mentioned) are significantly enriched in one of the sexes, suggesting that some of these RNAs might have sex-specific expression. Despite the number of differentially expressed mtRNAs being different across the clinical stages, most of these clusters overlap across the clinical stages, with six of them having a very reliable difference in their expression (*p* < 0.0001 with Wald test corrected using Benjamini and Hochberg method). These mtRNAs are mtRNA1, 33 nt long and encoded within the gene mt-tRNA Glutamic acid; mtRNA2, 32 nt long encoded within the gene mt-rRNA 12 S; mtRNA8 and mtRNA9 are isoforms of the same mt-sRNA with 3 nt of difference, 26–29 nt long and both encoded within the gene mt- tRNA Serine 2 (AGU/C); mtRNA16, 38 nt long encoded within the gene mt-tRNA Lysine; mtRNA57, 37 nt long encoded within the gene mt-COX2.

Although the six mtRNAs change in expression from the two initial clinical stages to the last is always significant, the type of change is not the same clusters (Figures 2C,D). Furthermore, only one of these RNA clusters was significantly different across sexes, while the others were unaffected. The first cluster, mtRNA1, shows a consistent decreasing trend that goes from being the highest during the treatment stage to intermediate value during the convalescence stage, and finally reaches the lowest during the rehabilitation stage. This suggests that somehow the SARS-CoV-2 infection can lower the expression of this mtRNA even after the infection is gone. The second cluster, mtRNA2, has similar expression during the first two stages, but it is upregulated during the last clinical stage, showing an opposite regulation from mtRNA1. Likewise, the clusters mtRNAs 16 and 57 are relatively stable during treatment and convalescence but upregulated during the rehabilitation stage. Interestingly, the type of gene (i.e., rRNA and protein-coding genes) where an mtRNA is encoded does not seem to affect whether it will be down- or upregulated during the rehabilitation stage. Nonetheless, clusters such as mtRNA 8 and 9, encoded within the same mt-tRNA, have similar patterns of expression. Indeed, as they differ only of 3 nucleotides, we can see that both downregulate only during the convalescence stage and have the highest expression at the rehabilitation stage. These results clearly suggest that the SARS-CoV-2 effect on the mitochondrial transcription is specific to singular mt-sRNAs rather than general.

**FIGURE 3:**
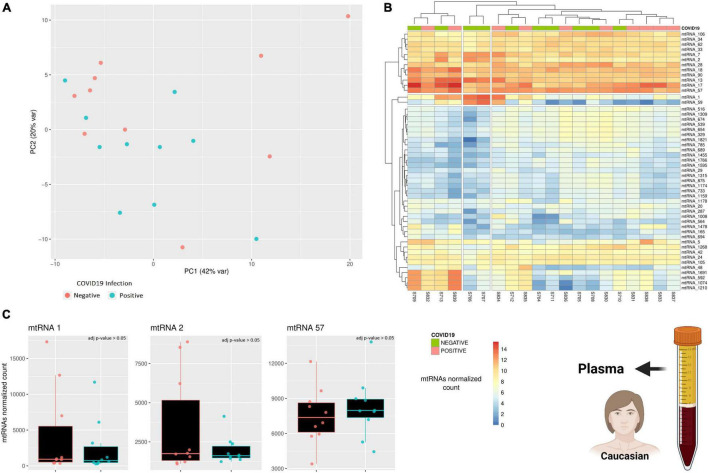
A comparison across of small RNAs expression between COVID-19 patients and age-matched healthy individuals. **(A)** The PCA shows how similar is the general small mitochondrial RNAs expression across patients and healthy individuals. The variability of each principal component is in the axis label. **(B)** The heatmap compare the expression of small RNAs encoded on the mitochondrial genome. The rows represent clusters of small RNAs (mtRNAs), while the columns are individuals. **(C)** The barplot compare the expression of several mtRNAs that are differentially transcribed across the three clinical stages. Each datapoint represent a different patient or healthy individual. The icons in the figure on the right-side highlights from which population and type of sample the data are generated. The icons have been made using Biorender.

Due to the limited amount of data, the results presented so far lack reliability, as we could only focus on a limited cohort and use only one sample type (PBMC). To try increasing the reliability of this study, a further dataset exploring the expression of small mitochondrial RNAs from a different sample type was included (Farr et al., 2021). This small RNA dataset includes plasma samples from 10 individuals positive for COVID-19 and 10 age-matched individuals negative for COVID-19, however, there is no information regarding the disease progression or severity. A PCA analysis was performed to verify if there is any small mitochondrial RNA able to discern between the two states (positive/negative), however, there was no clustering across the samples (Figure 3A). Similarly, performing a differential expression analysis showed that none of the small mitochondrial RNAs expression was significantly different in the individual infected (Figure 3B), suggesting that pooling together different stages of the infection—and recovery—can hide the changes in expression caused by the infection. Nonetheless, it is possible that the lack of significance in the small mitochondrial RNAs might be due to the change in sample type. Indeed, three of the six small mitochondrial RNAs mentioned in the previous paragraph are absent in these plasma samples (Figure 3C), suggesting an important role for tissue-specific regulation on the expression of these RNAs. The three small mitochondrial RNAs present in the sample have thousands of copies, like what was observed in the dataset, however, a direct comparison could be misleading. Indeed, the different protocols for the library preparation, the sequencing depth, and the initial amount of RNA would all be strong confounding factors. Nonetheless, these results suggest that the small mitochondrial RNAs of interest might be diverse across tissues and that separating COVID-19 patients according to their different stages of recovery would help to identify small mitochondrial RNAs of interest.

## Discussion

Considering together the results from canonical and non-canonical genes it can be concluded that SARS-CoV-2 infection does not affect general mitochondrial transcription but, rather, affects the biogenesis pathways of specific mitochondrial products. This effect seems to be more marked on the mt-sRNAs rather than longer ones, although the reason for such a pattern can only be speculated at this stage. Nonetheless, the analysis shows that the initial hypothesis was only partially correct. Indeed, while the differential expression analysis clearly shows that some mitochondrial non-canonical genes are disrupted by the SARS-CoV-2 infection, the disruption seems limited to a specific class of genes (mt-sRNAs) and is not extended to all mitochondrial canonical and non-canonical genes. Furthermore, the disruption detected in the first dataset was not confirmed in the second dataset, suggesting a more complex scenario.

The second dataset was included to verify if a similar disruption in the small mitochondrial RNAs expression would be present in a different sample type, thus testing if the RNAs of interest identified (e.g., mtRNA1 and 2) are likely to be upregulated through the body or only in the PBMC. However, plasma from the same samples is not available, and the second dataset changes multiple factors from the first. Indeed, in the second dataset, most individuals are women (60%) and, contrary to the previous cohort, they were recruited in central Europe, thus likely being of different ethnicity from the first dataset (Caucasian rather than Asian). Furthermore, different materials and methods were used to collect the samples and create the dataset. Thus, the two datasets differ in population, sampling and sequencing method, and tissue type, making it rather hard to know why the small mitochondrial RNAs expression is similar across COVID-19 patients and healthy individuals. However, the comparison between the datasets provides a different kind of insight.

As the data is limited, this study can only be preliminary, however, the results shown here can be used to find insights that can guide future experiments, where, hopefully, more definitive evidence can be found. The first insight we can gather by the comparison of the two small RNAs datasets is that the time of COVID-19 infection should be noted when comparing the samples, as mixing people at different stages might give misleading results. The second insight is that despite the tissue-specific expression of small mitochondrial RNAs, some of them is conserved across tissues (such as mtRNA1 and 2), thus it could be possible to easily test their abundance in many samples by qPCR and without needing to perform sequencing of each and every sample. Along the same lines, the third insight we can gather from the results is that due to the tissue-specific nature of these samples it would be important to gather samples from the tissue that better match the biology of the problem. For example, to study long COVID syndrome, having data from muscle tissues would be ideal, and if they were available, they would have been included in this study. The fourth insight we can gather is that sex might be an important variant in the expression of mitochondrial non-canonical genes, as being male or female affects the expression of some small mitochondrial RNAs in the small cohort here analyzed. The last insight that we can gather is that populations effects might be particularly relevant when studying the disruption of mitochondrial genes expression, as different mitochondria haplotypes might have different expression (as seen in fruit flies before). In conclusion, despite having only preliminary results, and in need of further experiments, it can be concluded that the mt-sRNAs expression can change during the recovery from COVID-19, making these relatively unknown RNAs good candidates for further studies into their role in long COVID.

## Materials and Methods

The long RNA datasets were downloaded from the NCBI database and then aligned to the known sequences of the three mt-lncRNAs using the aligner Bowtie2 (Langmead and Salzberg, 2013) using the –local setting, thus not requiring any adapter trimming. The count of each mt-lncRNA was computed using the tool featureCounts (Liao et al., 2014) ignoring multi-mapping reads (there were no multi-mapping reads). I used the gene count output to analyze the expression of mt-lncRNA through the package DEseq2 in R (Love et al., 2014). Similarly, all statistical tests were performed using DEseq2, which involves testing the differences across samples through a Wald test and correcting for multiple testing by using Benjamini and Hochberg method.

The small RNA datasets were downloaded from the NCBI database and then aligned to the mitochondrial reference genome (NC_012920.1) using the aligner Bowtie2 (Langmead and Salzberg, 2013) using the –local setting, thus not requiring any adapter trimming. The alignment files (.bam) were used to generate a sequence file (.fasta) including all the reads from the small RNA datasets that could be aligned to the mitochondrial genome. This file was then used to generate an assembly of all the small RNAs in the dataset by using Inchworm, the first tool of the Trinity package (Grabherr et al., 2011). Inchworm is a tool meant to be used on mRNA data, thus it tries to create an assembly with longer RNAs rather than short ones, so using this tool creates an assembly of small RNAs clusters, rather than identifying single small RNAs. Although not optimal, this approach should be accurate enough to enable at least a comparison between small RNA clusters (Bermúdez-Barrientos et al., 2020). The assembly of small RNA clusters was then used to map all the small RNAs mapping to the mitochondrial genome using the program ShortStack, which then computed the gene counts for each mt-sRNA cluster. I used the gene count output to analyze the expression of mt-lncRNA through the package DEseq2 in R (Love et al., 2014). Similarly, all statistical tests were performed using DEseq2, which involves testing the differences across samples through a Wald test and correcting for multiple testing by using Benjamini and Hochberg method. Statistics of the comparisons between clinical stages and sexes are available in Supplementary Table 1.

The differentially expressed mtRNAs mentioned sequences are the following:

mtRNA_1 UUGGUCGUGGUUGUAGUCCGUGCGAGAAU ACCA;

mtRNA_2 AUAGGUUUGGUCCUAGCCUUUCUAUUAGC UCU;

mtRNA_8 GAGAAAGCUCACAAGAACUGCUAACU;

mtRNA_9 ACGAAAGUGGCUUUAACAUAUCUGAACA;

mtRNA_16 CACUGUAAAGCUAACUUAGCAUUAACCUU UUAAGUUAA;

mtRNA_57 AGUAGCGUCUUGUAGACCUACUUGCGCU GCACCACCA;

## Data Availability Statement

The data used in the first part of this study are available under the accession PRJNA662985 in the NCBI database (https://www.ncbi.nlm.nih.gov/bioproject). There are 85 samples in the dataset, gathered from 18 individuals and separated into small and long RNA data. All the patients were hospitalized and enrolled between January-17-2020 and April-09-2020. All RNA samples were extracted from peripheral blood mononuclear cells (PBMC). Further information regarding the patients and the ethics of the study can be found in the article that first described the data (Zheng et al., 2020).

## Ethics Statement

The data used were already available online and validated by the human ethics committee of the university that first generated the data. The patients/participants provided their written informed consent to participate in this study.

## Author Contributions

AP conceived the study, performed all the analyses, made the figures, wrote, and reviewed the manuscript.

## Conflict of Interest

The author declares that the research was conducted in the absence of any commercial or financial relationships that could be construed as a potential conflict of interest.

## Publisher’s Note

All claims expressed in this article are solely those of the authors and do not necessarily represent those of their affiliated organizations, or those of the publisher, the editors and the reviewers. Any product that may be evaluated in this article, or claim that may be made by its manufacturer, is not guaranteed or endorsed by the publisher.
